# The Crisis at St. George's Hospital

**Published:** 1914-01-17

**Authors:** E. M. Wilson

**Affiliations:** Lieut.-Colonel. 15 Gilston Road, South Kensington


### THE EDITOR'S LETTER-BOX.

### The Crisis at St. George's Hospital.

*To the Editor of* THE HOSPITAL.

SIR,—I beg to express my entire concurrence in your article on St. George's Hospital in to-day's issue. I was present at the Special Court on December 19, and it seemed to me to be a great pity that the real and urgent necessities of the hospital, its present financial difficulties, and the uncertainty of its future prospects were entirely obscured by a personal question relating to the position of one individual. I think the vast majority of the governors, many of whom are old St. George's men, would agree that the present requirements and future policy of the hospital are of far greater importance than the claims of any one person, however valuable his services may have been, and I think your article should have the effect of driving away the confusion which undoubtedly existed among governors present at the meeting of December 19 as to the real business before the Court.—I am, Sir, your obedient servant,

E. M. WILSON, Lieut.-Colonel.

15 Gilston Road,
South Kensington,
January 10.

### Where is the Limit to Christmas Festivities?

*To the Editor of* THE HOSPITAL.

